# *Sarcocystis* spp. Investigations in Iberian Wild Caprinae Reveal a Remarkable Degree of Interconnection with Parasites’ Domestic Epidemiological Life Cycles

**DOI:** 10.3390/pathogens15080816

**Published:** 2026-08-01

**Authors:** Guillermo E. Delgado-De las Cuevas, Eglė Rudaitytė-Lukošienė, Petras Prakas, Josep Estruch, Roser Velarde, Antonio I. Plasencia-Gutiérrez, María L. García-Gil, Miguel Á. Habela, Rafael Calero-Bernal

**Affiliations:** 1 Animal Health Department, University of Extremadura, Avda. Universidad s/n, 10071 Cáceres, Spain; guillerdelc@gmail.com (G.E.D.-D.l.C.); mahabela@unex.es (M.Á.H.); 2 State Scientific Research Institute Nature Research Centre, Akademijos 2, 08412 Vilnius, Lithuania; egle.rudaityte@gmail.com; 3 Wildlife Ecology & Health Group (WE&H) and Servei d’Ecopatologia de Fauna Salvatge (SEFaS), Departament de Medicina i Cirurgia Animals, Facultat de Veterinària, Universitat Autònoma de Barcelona (UAB), 08193 Bellaterra, Spain; josep.estruch@uab.cat (J.E.); roser.velarde@uab.cat (R.V.); 4 Conselleria d’Agricultura, Pesca i Alimentació, Gobierno de las Islas Baleares, Calle Eusebi Estada 145, 07009 Palma, Spain; aiplasencia@fogaiba.caib.es; 5 Spanish National Electron Microscopy Centre, Avenida Complutense s/n, 28040 Madrid, Spain; 6 SALUVET Group, Animal Health Department, Complutense University of Madrid, Ciudad Universitaria s/n, 28040 Madrid, Spain

**Keywords:** sarcocysts, *18S rRNA*, *cox1*, molecular characterisation, morphology, phylogeny

## Abstract

Many uncertainties exist on the epidemiology of the *Sarcocystis* species infecting wild ruminants. In this study, tongue and/or diaphragm tissues of free-ranging wild Caprinae species (54 Pyrenean chamois, 52 mouflons, 146 Iberian ibexes, and 19 Balearean wild goats) residing in the mountain areas of Spain were selected for *Sarcocystis* spp. detection and species identification. By light microscopy, *Sarcocystis* sarcocyst frequency rates were considerably high (85.7% in chamois, 82.7% in mouflon, 52.7% in Iberian ibexes), except for the Balearean wild goat (5.3%), which was investigated for the very first time. One excised sarcocyst of Iberian ibex was subjected to transmission electron microscopy, revealing a sibling *S. capracanis* ultrastructure. Genomic DNA isolated from 57 sarcocysts excised from the hosts’ tissues was investigated through PCR amplicon sequencing of the *18S* rRNA and *cox1* genes. As a result, sequences of *S. capracanis*, *S. cornagliai*, *S. rossii* and *S. tenella* were detected, sharing high identity with conspecific sequences. Phylogenetic analyses confirmed a remarkable clustering of all *Sarcocystis* species in clades encompassing species transmitted via canids, except *S. cornagliai*, which is allocated in a clade with species using corvid birds as their definitive hosts. Therefore, a significant degree of interconnection between sylvatic and domestic *Sarcocystis* epidemiological cycles can be drawn in the study area.

## 1. Introduction

Cyst-forming coccidians of the genus *Sarcocystis* (Apicomplexa) constitute a large group of more than 240 species with an obligate two-host life cycle whose principal intermediate hosts (IHs) are herbivores, whilst carnivores act as their definitive hosts (DHs). *Sarcocystis* species develop their sexual reproduction in the intestine of DHs; consequently, oocysts/sporocysts are shed in their feces, serving as sources of infection for IHs. Once ingested, asexual multiplication of the parasites occurs primarily in the endothelial layers of blood vessels and finally merozoites convert to bradyzoites within tissue cysts; mature tissue cysts located mostly in striated musculature constitute the chronic stage of infection and stay latent until a specific DH preys on such host tissues to complete the life cycle [1].

It is mostly accepted that *Sarcocystis* species have a high specificity for their IH hosts, but recent findings have demonstrated a looser degree of specificity [1,2]. Although wild ruminants have been frequently investigated as IHs of *Sarcocystis* spp. (reviewed in [1]), the high prevalence of sarcocysts and the remarkable species diversity stand out as two factors that hinder efforts to unravel the species’ epidemiology. The available literature on *Sarcocystis* spp. infections in wild mountain ruminants (Caprinae) in Europe is summarized in Table S1 [3,4,5,6,7,8,9,10,11,12,13,14,15,16,17,18,19,20,21,22]; most studies only reported their presence at the genus level, and very few performed ultrastructural examination combined with molecular analyses. Although no report on zoonotic *Sarcocystis* spp. presence in wild ruminants has been published to date, food poisoning associated with *Sarcocystis* presence in big game meat has been communicated worldwide (reviewed in [1]); additionally, a One Health approach to the investigation and control of *Sarcocystis* has limited impact given the fact that species cycling does not comprise all three compartments of the principle.

Few studies dealing with *Sarcocystis* spp. presence in mountain ungulates in Spain have been carried out; only two of them attempted species identification by using molecular and/or electron microscopy methods (Table S1). Although distribution areas and ranging altitudes above 1000 m may differ from those of domestic animals [23,24,25], hosts that have also been scarcely investigated in the study area [1], potential interaction with extensively raised livestock and potential pathogenicity of some *Sarcocystis* spp. justify the present investigation. This study aimed at estimating the frequency of *Sarcocystis* infections in Balearean wild goat (*Capra hircus var. majorcan*/*Capra hircus aegagrus*); Pyrenean chamois (*Rupicapra pyrenaica pyrenaica* and *R. pyrenaica parva*), Iberian ibex (*Capra pyrenaica*) and mouflon (*Ovis orientalis musimon*), along with the identification of the species present, and ultimately to infer their phylogenetic relationship with previous genetic variants detected in other hosts in the same epidemiological scenario.

## 2. Materials and Methods

### 2.1. Sample Collection

**Figure 1:**
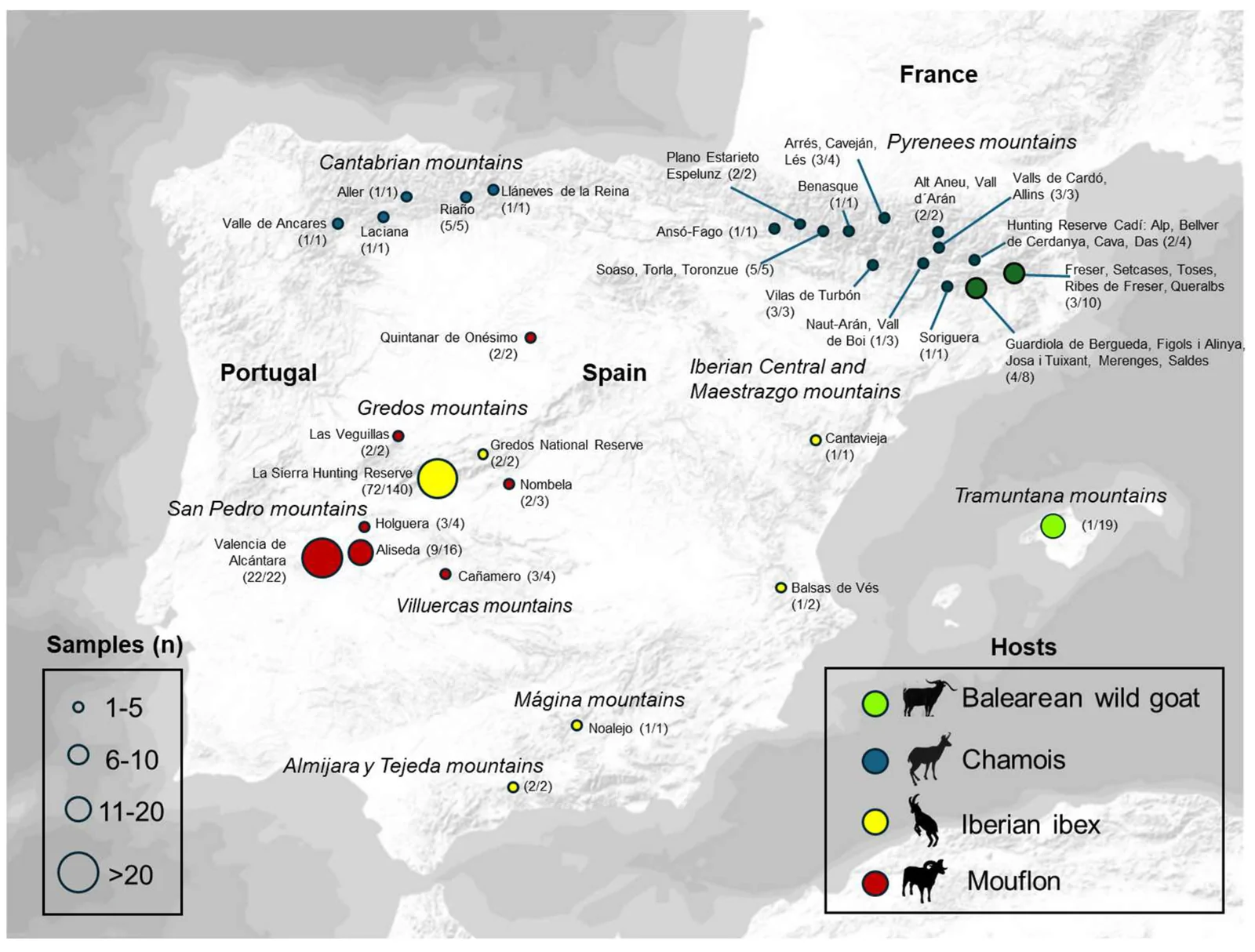
Wild Caprinae host species under study and sampling areas in Spain. Circle areas indicate the number of individuals sampled in each site. Data between parentheses indicate *Sarcocystis*-positive animals detected by light microscopy methods (compression smears and histology) among the total animals sampled in each sampling area.

Tongue and/or diaphragm muscles were obtained from 19 Balearean wild goats during the years 2019 to 2021, 54 Pyrenean chamois between 2006 and 2019, 146 Iberian ibexes from 2015 to 2020, and 52 mouflons from 2015 to 2018. Animals were legally hunted in different areas of Spain or found dead within the passive surveillance program for wild game species with causes unrelated to protozoan infection. Tissues were collected immediately after the hunt during veterinary inspection of carcasses or necropsy (Table 1; Figure 1) and shipped (refrigerated with ice packs) to the Animal Health Department at the Veterinary Faculty of the University of Extremadura (Cáceres, Spain), where they were stored frozen at −20 °C until further analyses. A set of direct detection methods was selectively used aiming to maximize sarcocyst (and zoite) detection for further molecular analyses. Prevalence estimates were not among the initial aims given the opportunistic sampling and the lack of sample size calculations.

### 2.2. Light Microscopy (LM) Examinations

Muscle samples were gently thawed at 4º C and processed as follows. Pieces of muscles were squashed between glass slides with 1 to 2 drops of saline solution to observe the presence of *Sarcocystis* by optical microscope (×10, ×40 magnifications) [26]. Eclipse Ci-L optical microscope equipped with a DS-Fi2 digital camera and the NIS-Elements software suite v.5.11 (Nikon, Tokyo, Japan) was used. Sarcocysts were excised from the muscle fibers using hypodermic needles and kept in 1.5 mL plastic vials containing 90% ethanol or 2.5% glutaraldehyde solution for DNA characterization or ultrastructure examinations, respectively. Moreover, for histological examination, 1 cm^3^ tissue portions were fixed in 4% buffered formalin and embedded in paraffin. Five-micrometer-thick sections were stained with hematoxylin and eosin (H & E) and observed under LM. Intensity of infection was evaluated as the number of *Sarcocystis*-like sarcocysts per cm^2^ of tissue section.

### 2.3. Transmission Electron Microscopy (TEM) Examinations

One excised sarcocyst belonging to Iberian ibex (code 19CM10) was processed as described in [27]. Briefly, samples were post-fixed, sectioned, and finally contrasted with 4% uranyl acetate and 3% lead citrate. Ultra-thin sections were examined at the Spanish National Centre for Electron Microscopy (Madrid, Spain), using a JEOL JEM 1400 Plus (JEOL Ltd., Tokyo, Japan) device at 80 kV.

### 2.4. Enzymatic Digestion of Samples

Initially, the digestion method was used only in one individual Iberian ibex (19CM10) and 19 Balearean wild goats, in order to improve sarcocyst detection and further DNA availability. Briefly, trypsin digestion of 5 gr of tongue muscular tissue, gently minced, in 50 mL of digestion solution (porcine trypsin 1% (Merck KGaA, Darmstadt-Germany), 0.5 gr, 40 U/mg), in sterile phosphate-buffered saline (PBS), in a stirring incubator, at 37 °C for 1 h, was followed by filtering the solution twice and centrifuging at 2400 rpm, 10 min, 4 °C; the supernatant was discarded and resuspended in saline solution and finally centrifuged in the same conditions, following the protocol described in [26]. Then, the pellet was conserved refrigerated in 90% ethanol until DNA extraction. The same procedure was followed for the muscle specimens of the chamois provided by the team of the UAB; in this case, only 2 gr of each specimen was available (Table 1).

### 2.5. Statistical Analyses

The Clopper–Pearson method was used to calculate the prevalence 95% confidence intervals due to the relatively small sample size. Contingency tables, utilizing Chi-squared tests, were performed to evaluate parasite burdens. Results were considered significant at *p* < 0.05. The SPSS software (version 29.0.1.0, IBM Corp., Armonk, NY, USA) was employed.

### 2.6. Molecular Analyses

Genomic DNA was extracted from excised sarcocysts, specifically 3 from 1 Balearean wild goat, 17 from 7 Pyrenean chamois, 24 from 19 Iberian ibexes, and 23 from 16 mouflons (Table 1), using the QIAamp^®^ DNA micro kit (Qiagen, Hilden, Germany), according to the manufacturer’s instructions. Partial *18S* rRNA sequences (1823–1831 bp long portion) were amplified using overlapping primer pairs SarAF/SarBR (1075 bp) and SarCF/SarDR (950 bp) for most of the samples, while the SUNIF3/SUNIR2 (940 bp) primer pair was additionally used with samples from feral goat [28,29,30]. Mitochondrial cytochrome C oxidase subunit I gene (*cox1*) sequences were amplified with the SF1 forward primer in combination with one of the following reverse primers: SR8D (1029 bp) [16,31], SR11 (1077 bp) [32], or SR12H (907 bp) [33]. Two primers (StecagrliF: 5′-GAATACGGAGGCCGTTGACT-3′; StecaR: 5′-CCAAATCCGGGCAATATTAAA-3′) were developed and used for sequencing selected samples that initially yielded incomplete or poor-quality sequences, in order to obtain full-length sequences. PCR reactions were performed using the DreamTaq PCR Master Mix (Thermo Fisher Scientific Baltics, Vilnius, Lithuania). The cycling conditions started for 5 min at 95 °C, followed by 35 cycles of 45 s at 94 °C, 60 s at 54–60 °C, depending on the primer pair and 80 s at 72 °C, followed by a final extension step at 72 °C for 5 min. PCR products were evaluated using 1.5% agarose gel electrophoresis and purified with exonuclease EXOI and alkaline phosphatase Fast AP (Thermo Fisher Scientific Baltics, Vilnius, Lithuania). All sequences generated in this study have been deposited in GenBank (NCBI) under accession numbers PZ406984–PZ407047 (*18S* rRNA gene) and PZ436359–PZ436426 (*cox1* gene). The sequences obtained were then compared with those of *Sarcocystis* spp. using the online Nucleotide BLAST program (BLASTn algorithm) (http://blast.ncbi.nlm.nih.gov/, accessed on 22 April 2026).

### 2.7. Phylogenetic Analyses

The resulting sequences were imported into MEGA12 software [34], where they were inspected and manually edited if necessary. Multiple sequence alignments were generated using the MUSCLE algorithm [35]. Appropriate nucleotide substitution models were selected using TOPALi v2.5 software [36], which was also used for Bayesian phylogenetic inference. The final *18S* rRNA dataset consisted of 187 sequences with 2054 aligned nucleotide positions. The best-fitting model was HKY+I+G. The cox1 dataset included 201 sequences and 889 aligned nucleotide positions, with SYM+I+G selected as the optimal model. To avoid excessive dataset size, up to five representative sequences per selected species were included in the analysis, together with ten sequences each of *S. tenella* and *S. capracanis*. The outgroup taxa included *Cystoisospora belli* (AB268326), *Toxoplasma gondii* (PZ112984), and *Eumonospora henryae* (LC595644) for the *18S* rRNA dataset, and *Hammondia heydorni* (JX473250), *Neospora caninum* (JX473252), *T*. *gondii* (KT363924), and *Eimeria iyoensis* (LC806970) for the *cox1* dataset. Among the sequences obtained in this study (Table 2), only distinct haplotypes were retained for phylogenetic analysis, yielding 25 unique *18S* rRNA sequences and 64 unique *cox1* sequences.

## 3. Results

### 3.1. Occurrence of Sarcocysts in Muscle Tissues of Iberian Wild Caprinae

By using compression smear, different degrees of infection frequency were observed: 85.7% (18/21) in Pyrenean chamois, 82.7% (43/52) in mouflon, 52.7% (77/146) in Iberian ibex, and 5.3% (1/19) in Balearean wild goat (Table 1). The frequency of *Sarcocystis* spp. infections varied significantly among the four investigated Caprinae hosts (Χ^2^ = 43.09, df = 3, *p* < 0.001). Post hoc analyses using adjusted standardized residuals reveal different infection patterns, with mouflon being the most susceptible to infection by *Sarcocystis* (residual = 4.02), followed by chamois (residual = 2.66); however, Spanish Ibex showed lower parasitic infection (residual = −2.23), and the boc the minimal (residual = −4.90).

In addition, different cyst burdens were observed among the investigated hosts (Table 1); notably, in descending order, 3.93, 2.34, 1.29, and 0.67 sarcocysts/cm^2^ were recorded in mouflon, chamois, Iberian ibex, and Balearean wild goat, respectively. A Kruskal–Wallis test showed significant differences in parasite burden among the four host species (χ^2^ = 10.12, df = 3, *p* = 0.02, ε^2^ = 0.092).

### 3.2. Morphological Characterization of Specimens

**Figure 2:**
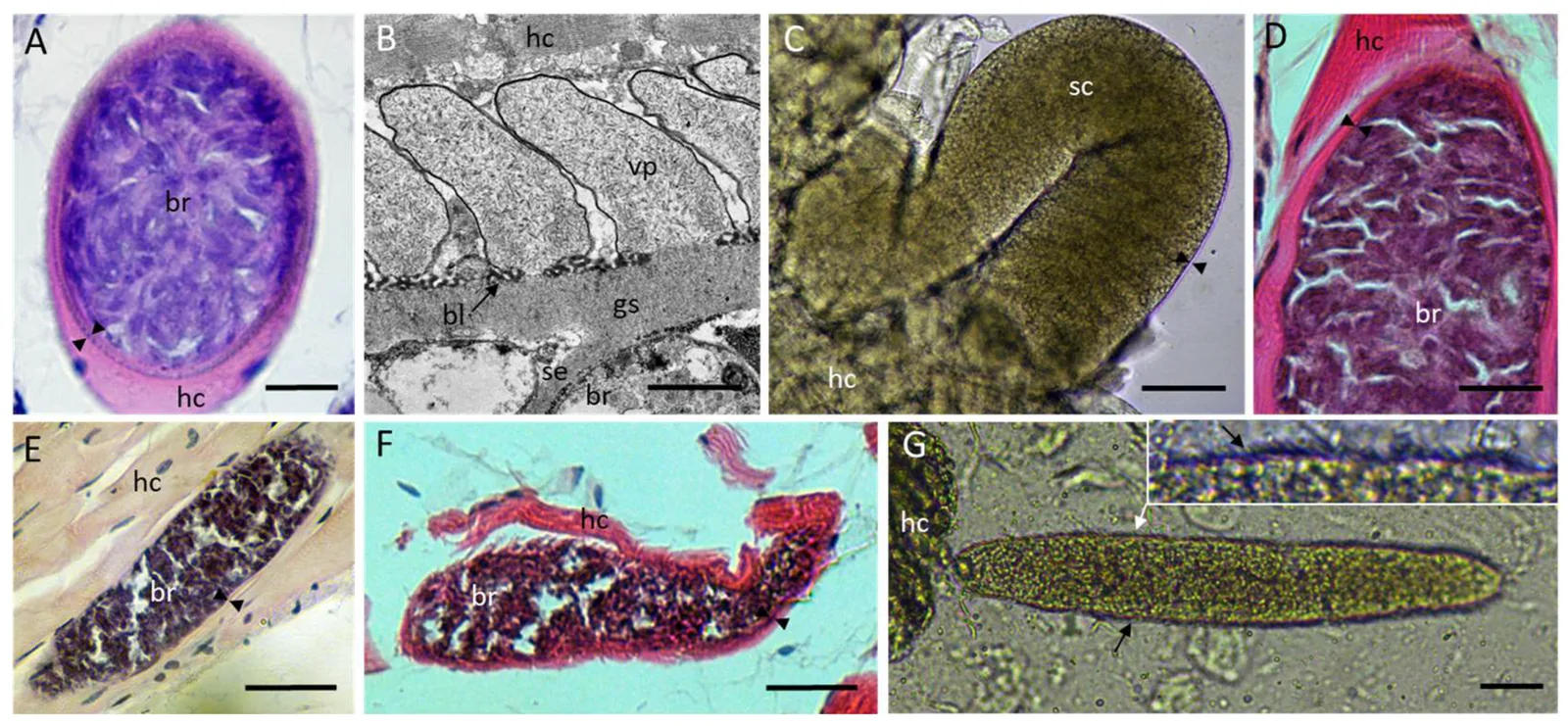
**Micrographs of *Sarcocystis* sarcocysts detected in the Iberian wild mountain ungulates.** (**A**) Thick-walled sarcocyst of Iberian ibex 19CM10. H&E staining. (**B**) Ultrastructure of the *Sarcocystis capracanis* sarcocyst in Iberian ibex 19CM10. TEM. (**C**) Apparently, thin-walled sarcocyst of Pyrenean chamois 17SA1. Unstained. (**D**) Thin-walled sarcocyst of Pyrenean chamois 19SA1. H&E staining. (**E**) Thin-walled sarcocyst of mouflon 17MU6. H&E staining. (**F**) Apparently thin-walled sarcocyst of a degraded sarcocyst of Balearean wild goat 19BOC1. H&E staining. (**G**) Finger-like villar protrusions (arrows) resembling those of *S. cornagliai* are visible at the surface of a sarcocyst of Balearean wild goat 19BOC1 excised from the muscle tissue. Unstained. Br: bradyzoites; hc: host-cell; bl: blebs; gs: ground substance; se: septae; vp: villar protrusion; sc: sarcocyst. Note: opposing arrowheads point at the cyst walls. Scale bars (**A**,**D**): 20 µm; B: 1 µm; (**C**,**F**,**G**): 50 µm; E: 25 µm.

Unlike in specific cases (Figure 2A), mostly thin-walled sarcocysts were observed by histopathological examination (Figure 2); nevertheless, fresh examination of the tissues in search of specimens for further individual analyses revealed up to 1100 μm long sarcocysts with clear 4 to 6 μm long finger-like or palisade-like villar protrusions (vp) resembling those of *S. cornagliai* in the 19BOC1 specimen of Balearean wild goat.

Additional effort was made for the Iberian ibex 19CM10, for which a cyst could be examined ultrastructurally (Figure 2B). A thick-walled *S. capracanis*-like sarcocyst presenting typical 3 μm long, tightly packed, and cylindrical-shaped vp of the TEM Type 14 [1] was observed.

### 3.3. Species Identification and Molecular Characterization

Molecular identification based on *18S* rRNA and *cox1* genes revealed the presence of different *Sarcocystis* species across the examined hosts. In chamois, *S. tenella* was identified based on 15 *18S* rRNA sequences (1828–1829 bp) and 17 *cox1* sequences (1005–1029 bp). In mouflon, *S. tenella* was detected based on 23 sequences for both *18S* rRNA (1828–1829 bp) and *cox1* (907–1029 bp). In Iberian ibex, *S. capracanis* was the predominant species, represented by 22 *18S* rRNA sequences (1823–1827 bp) and 24 *cox1* sequences (1029 bp). In addition, a single *18S* rRNA and *cox1* sequence of *S. rossii* (1831 bp and 1029 bp, respectively) was obtained from this host. In Balearean wild goat, *S. cornagliai* was identified based on three sequences for each locus, with *18S* rRNA sequences of 1874 bp and *cox1* sequences of 1077 bp. All sequence similarity values, including intraspecific variation, similarity to GenBank sequences of the same species, and the closest related species, are summarized in Table 2. Overall, the results demonstrated host-associated distribution of *Sarcocystis* species, with consistent identification supported by both genetic markers.

### 3.4. Phylogenetic Analyses

**Figure 3:**
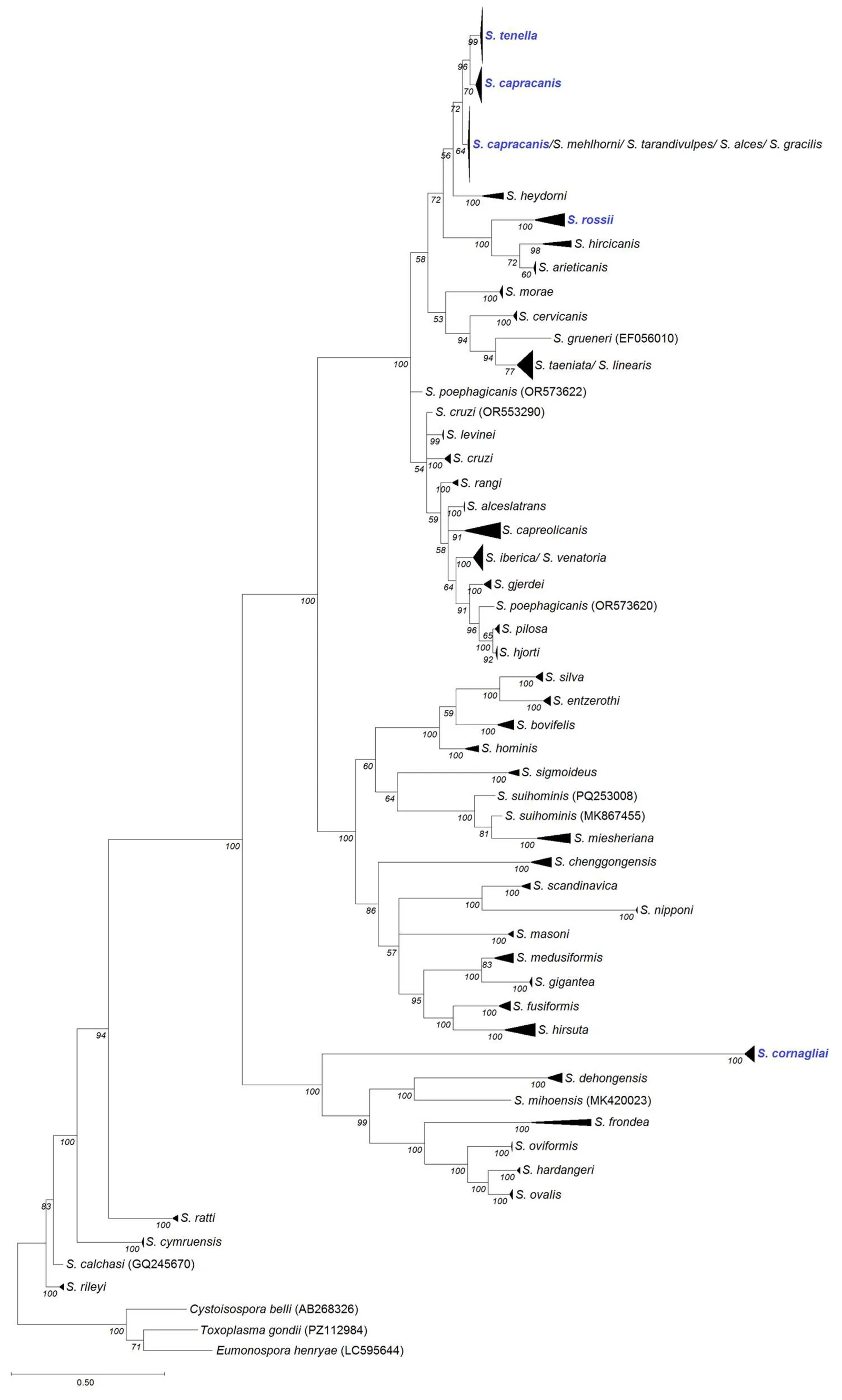
Phylogenetic analysis based on the *18S* rRNA gene of the *Sarcocystis* spp. (*S. capracanis*, *S. cornagliai*, *S. rossii*, and *S. tenella*) detected in the wild Caprinae hosts investigated in Spain. Triangles represent collapsed groups of taxa used to simplify tree visualization.

**Figure 4:**
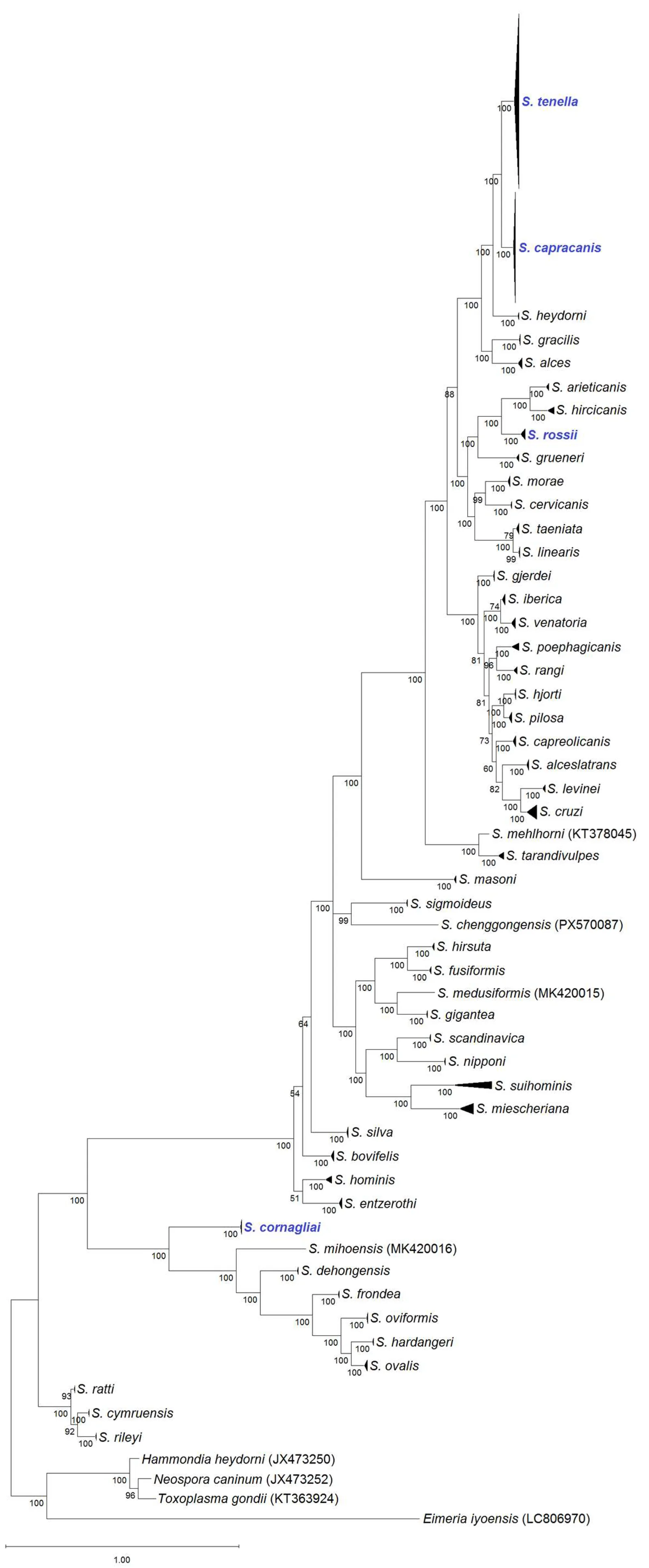
Phylogenetic analysis based on the *cox1* gene of the *Sarcocystis* spp. (*S. capracanis*, *S. cornagliai*, *S. rossii*, and *S. tenella*) detected in the wild Caprinae hosts investigated in Spain. Triangles represent collapsed groups of taxa used to simplify tree visualization.

Phylogenetic trees were constructed using both molecular loci, the *18S* rRNA gene and *cox1* (Figure 3 and Figure 4). The *18S* rRNA gene sequences were not sufficiently variable to discriminate some *S. capracanis* sequences from those of *S. mehlhorni*, *S. tarandivulpes*, *S. alces*, and *S. gracilis*. In addition, in the phylogenetic tree composed of *18S* rRNA sequences, some other species, including *S. taeniata*, *S. linearis*, *S. poephagicanis*, *S. cruzi*, *S. iberica* and *S. venatoria*, could not be clearly distinguished from their closely related species. In the *18S* rRNA phylogeny, *S. tenella* sequences grouped together with sequences of the same species. Based on *cox1* phylogeny, *S. tenella* and *S. capracanis* each formed distinct monophyletic clusters and were placed as sister taxa (Figure S2). Across both phylogenies, *S. rossii* clustered with sequences of the same species and formed a sister group to the clade comprising *S. arieticanis* and *S. hircicanis*. In both trees, *S. cornagliai* formed a monophyletic clade and was closely related to several wild ruminant-related *Sarcocystis* species, including *S. dehongensis*, *S. mihoensis*, *S. frondea*, *S. hardangeri*, *S. ovalis*, and *S. oviformis*.

## 4. Discussion

Two common *Sarcocystis* spp. for the Caprinae subfamily hosts (*S. capracanis* and *S. tenella*) and two rare ones (*S. cornagliai* and *S. rossii*) were detected by means of molecular analyses in the Iberian wild mountain ungulates. Specifically, *S. tenella* was identified in Pyrenean chamois and mouflon, *S. capracanis* and *S. rossii* in Iberian ibex, and *S. cornagliai* in Balearean wild goat.

This is the first report of *Sarcocystis* spp. infection in the Balearean wild goat, a Caprinae introduced in the Majorca Island by during the Neolithic period (2050–2300 years BC) at a very initial domestication stage. Shortly after, individuals became free and occupied the ecological niche of the extinct Caprinae *Myotragus balearicus* [37]. Low *Sarcocystis* occurrence and cyst burden identified might be related to the low exposure to the parasite.

Findings of *S. cornagliai* were unexpected and add a new host record for the species that, to the authors’ knowledge, had until date only been reported in Alpine ibex (*Capra ibex*) and Alpine chamois (*Rupicapra rupicapra*) and never outside the Alpine territories of Austria, Italy and Germany [5,17,22]. The detection of *S. cornagliai* also suggests a corvid-related life cycle since it is placed in a well-supported group (Figure 4) encompassing *S. dehongensis*, *S. frondea*, *S. ovalis*, and *S. oviformis* having corvids as DHs [38,39,40]. *Sarcocystis ovalis* from moose (*Alces alces*) is transmitted via the omnivorous Corvidae magpie (*Pica pica*) [40], and this might be the case for the *S. cornagliai* identified herein where an isolated environment and rugged terrain in the Tramuntana mountains of the Majorca Island may explain the limited circulation of *Sarcocystis* species. As other *Sarcocystis* species are expected to parasitize Balearean wild goats, additional efforts are warranted.

In Pyrenean chamois, a *Sarcocystis* species (*S. tenella*) has been identified for the first time, and the frequency of detection was relatively high (93.8%, n = 48), in accord with figures reported in the Alpine chamois in Germany (78.5%, n = 181) [10], Italy (79.8%, n = 198) [3], and Slovakia and Poland (100%) where few animals were tested (n = 6 and n = 3, respectively) [8,11] (Table S1). Herein, only the domestic sheep-related species *S. tenella* was detected infecting chamois from the Northern Spanish territories; such a finding is in accord with the pioneer study [3] that reported two ultrastructurally different types of sarcocysts, one resembling *S. tenella*/*S. capracanis* and the other showing mushroom-like vp; the second species was tentatively described and named as *S. cornagliai* [5]. Therefore, under the view of the available literature, other *Sarcocystis* species might be circulating in the investigated animals, and additional samplings will add valuable information in this regard. Low genetic diversity is observed between the *S. tenella* isolates detected in Pyrenean chamois for *18S rRNA* gene (Table 2). However, this diversity increased at the *cox1* gene when it was compared intraspecifically with other ruminant-derived isolates. One study on *Sarcocystis* in domestic sheep in Spain reported high haplotype intraspecific diversity, with variations up to 20 nucleotide positions, but the majority were silent mutations [29]. In Pyrenean chamois, nucleotide variation was similarly high, up to 15 positions. Equally, there was a relatively high number of haplotypes (17), and the intraspecific diversity was elevated up to 4.5%, even surpassing the figures reported in sheep [29]. Conversely, low genetic diversity for the species *S. tenella* was also confirmed for isolates infecting Tatra chamois (*Rupicapra rupicapra tatra*) in Poland, showing 99.23% identity with domestic sheep isolates *S. tenella* at the *cox1* gene [11].

The occurrence of *Sarcocystis* spp. in the Iberian ibexes studied herein reached remarkable figures (52.7%); nevertheless, higher frequencies of detections have been previously detected in the same host in Spain (84%) [21], and in other close ibex species like the Alpine ibex (*Capra ibex*) (86%) in Italy [17].

In a previous study carried out in Iberian ibex from Southwestern Spain, *S. hircicanis* was suggested because of observation of thin and smooth cyst wall by light microscopy; however, molecular and ultrastructural analyses were not performed [21].

With the aid of molecular analyses, in most positive samples, *S. capracanis* was detected, and in one animal (19CM10), the uncommon species *S. rossii* [22] was found. This prompted us to make an additional effort to try to characterize the organism by TEM given the fact that at that moment, the species *S. rossii* was still undescribed. Details observed (Figure 2A) agreed with the morphology described for *S. capracanis* in Alpine ibex [17,22]. Low cyst burden detected in such specimen (1.2 sarcocysts/cm^2^) hampered the examination of additional sarcocysts by TEM. The recently identified *S. rossii* exhibited minimal variability at the *cox1* gene, with the same species observed in Alpine ibex (1.6%). However, this variability significantly increased in interspecific comparisons, with the closest relative, *S. hircicanis*, displaying diversity up to 13.8%. The intraspecific variability of *S. capracanis* was 2.7%, paralleling the diversity observed in the sister species *S. tenella*. The closest interspecific comparison was with *S. tenella*, where the diversity ranged between 2.6 and 8.6%, but this was comparatively minor when juxtaposed with the diversity of *S. rossii*. Another cryptic species, *S. capricornis*-like, was described only ultrastructurally as by Cornaglia et al. [17] in one specimen of Iberian ibex. It exhibited characteristic indented edges of finger vp (< 2.8 μm long and <0.75 μm wide), with an axial fibrillar core running through the middle of vp (CW type 41 [1]). No other research has noticed this peculiar *Sarcocystis* spp., and therefore additional investigations on the Iberian wild goat are justified, aiming at potential involvement of other *Sarcocystis* spp.

A very high infection frequency was observed in mouflon (82.7%), as in other studies from additional European countries like Austria, Slovak Republic or Germany (86.5–100%) [13,14,16]. Molecular analyses confirmed *S. tenella* as the unique species present. A *S. tenella*-like organism was ultrastructurally examined and reported in mouflon in Italy [12], and its presence was also confirmed in Germany [13], and in several other hosts like argali (*Ovis ammon*) in China [41], Tatra chamois (*Rupicapra rupicapra tatrica*) in Poland [11], and Barbary sheep (*Ammotragus lervia*) in Spain [2]. Differences in *S. tenella* isolates from mouflon and chamois were negligible (< 1.5%) and might be explained by their habitat overlap, especially during winter periods. As monospecific infections by *S. tenella* seem to be common in mouflon, a potential close contact with domestic sheep is suggested; the pathogenic potential of *S. tenella* in mouflon still remains to be elucidated.

Similarly, in the wild Caprinae Barbary sheep (*Ammotragus lervia*) from Spain, only *S. tenella* and *S. capracanis* species were found [2]; there is an apparent limited diversity of *Sarcocystis* spp. infecting wild mountain ungulates/Caprinae of Spain, in contrast with what is commonly observed in other Cervidae hosts like red deer (*Cervus elaphus*) [31], roe deer (*Capreolus capreolus*) [33,42,43,44,45,46], and fallow deer (*Dama dama*) [47,48,49], ranging free in Spain. The variability in *S. capracanis* and *S. tenella*, should be further studied to unravel the potential influence of geographical factors rather than dependency on the *Sarcocystis* species, as was previously suggested in domestic sheep [29,50].

While *S. tenella* and *S. capracanis* were especially pathogenic to domestic sheep and goats respectively, with remarkable clinical signs (e.g., abortions) observed [1], additional effort is needed to unravel the potential pathogenic role of the genus for the wild ruminant species studied.

Some limitations have been identified in the present study, namely the following: (a) given the opportunistic sampling, the small sampling size for some of the hosts hampered the precise estimation of prevalence figures; (b) diagnostic methods have not been systematically used for all collected samples, making host-to-host comparisons difficult; and (c) only one sarcocyst was examined by TEM and two molecular markers used to characterize the organisms, so additional efforts would have been provided a more accurate characterization of the *Sarcocystis* species detected.

Despite the limitations reported above, the present paper adds complementary data to the complex epidemiology of the *Sarcocystis* genus and provides additional evidence of the lack of specificity of some *Sarcocystis* species for their Caprinae intermediate hosts.

## 5. Conclusions

A low diversity of *Sarcocystis* species infecting wild Caprinae observed in Spain might be linked to the fact that the sheep-related *S. tenella* and goat-related *S. capracanis*, both with Canidae as DHs, predominate, and repetitive infections may result in higher cyst loads than other species, possibly hampering the detection of minority (or more host-restricted) *Sarcocystis* spp. Indeed, there is a clear interconnection of domestic–sylvatic epidemiological cycles of the species, which might interfere with health programs. Identification of rare species like *S. cornagliai* and *S. rossii* has allowed us to expand the host range of both species and also justifies additional surveys for potential undescribed species detection in such hosts. Finally, further research based on the analysis of complementary genetic markers in these *Sarcocystis* spp. is warranted to determine their true host-specificity and, in absence of animal experimentation, to achieve the inference of their most plausible DHs in the study area.

## Figures and Tables

**Table 1:** Muscle tissues collection, frequency of tissue-cyst detection and morphological features of *Sarcocystis* spp. sarcocysts in wild Caprinae in Spain.

Intermediate Hosts				
Hosts	Balearean wild Goat (*Capra hircus var. majorcan*)	Pyrenean Chamois (*Rupicapra pyrenaica*)	Iberian Ibex (*Capra pyrenaica*)	Mouflon (*Ovis orientalis musimon*)
Direct detection				
Compression smear (%, 95% CI, *n* positive/n examined)	5.3% (0.13–26.03%) (1/19)	85.7% (63.66–96.95%) (18/21)	52.7% (44.32–61.05%) (77/146)	82.7% (69.67–91.77%) (43/52)
H&E staining (%, n positive slides/*n* tested slides)	66.7% (2/3)	93.8% (45/48) ^b^	28.2% (42/149)	46.7% (14/30)
Sarcocyst burden (*n* cysts/cm^2^, 95% CI)	0.67 (−0.16–1.50; *n* = 5)	2.34 (1.30–3.39; *n* = 45)	1.29 (0.94–1.65; *n* = 38)	3.93 (1.84–6.02; *n* = 23)
Trypsin digestion (cysts or bradyzoites observed)	10.5% (2/19)	ND ^c^	Only done for 19CM10, with positive results.	ND
Morphological evaluation by light microscopy examination	Thick-walled, with sloped finger vp by impression smear	Palisade thin-walled, 0.8 μm or minor	Palisade thin-walled, up to 2.1 μm	Palisade thin-walled, minor 1.5 μm or imperceptible
Sarcocysts length & width means (μm)	63.2 ± 43.63 × 28.4 ± 6.25 (*n* = 5) (range: 144 × 36–26 × 22)	116.83 ± 18.04 × 49.69 ± 4.35 (*n* = 101) (range: 408 × 128.1–23.1 × 18.9)	82.94 ± 14.16 × 38.06 ± 2.73 (*n* = 114) (range: 562.1 × 76–23.1 × 10.5)	79.32 ± 9.17 × 31.47 ± 1.71 (*n* = 228) (range: 462 × 100–10.5 × 10.5)
Sarcocysts isolated for molecular analyses (*n*)	3 ^d^	17 ^e^	24 ^f^	23 ^g^

Note: Sampling areas are shown in Supplementary Figure S1. ^b^ Thirty-three individuals were solely investigated by H&E staining at the Autonomous University of Barcelona by one of us (J.E.). ^c^ Skeletal muscle tissues of 66 Pyrenean chamois (each piece was 2 gr) were analyzed by trypsin digestion; 22 pools of 6 g were digested and observed under light microscopy; bradyzoites but no cysts were observed only in one pool; 30 µL of each digest was subjected to DNA extraction and *cox1* amplification using SF1-SR5 primers as indicated in the present paper. Sanger sequencing of amplicons (n = 3) resulted in unreadable lectures. 95% CI: confidence interval at 95%. ND: not done. ^d^ Balearean wild goat specimens: 19BOC1.1, 19BOC1.2, 19BOC1.3. ^e^ Pyrenean chamois specimens: 16SA2.1, 16SA3.1, 16SA4.1, 16SA5.1, 16SA7.1, 16SA8.1, 17SA1.1, 17SA1.2, 17SA1.3, 18SA1.2, 18SA3.1, 18SA3.9, 18SA4.1, 18SA4.2, 19SA1.1, 19SA2.1, 19SA4.1. ^f^ Iberian ibex specimens: 17CM2.1, 17CM2.4, 18CM1.1, 18CM3.1,18CM4.1, 18CM5.2, 18CM6.1, 18CM7.1, 18CM11.1, 19CM3.2, 19CM3.5, 19CM4.2, 19CM5.4, 19CM7.8, 19CM9.1, 19CM10.2, 19CM11.4, 19CM12.2, 19CM14.1, 19CM15.1, 19CM15.6, 20CM1.5, 20CM2.5, 20CM2.8. ^g^ Mouflon specimens: 16MU1.1, 16MU2.1, 16MU3.1, 16MU4.1, 17MU2.1, 17MU3.1, 18MU1.1, 18MU2.1, 18MU3.1, 18MU5.1, 18MU6.1, 18MU7.1, 18MU8.1, 18MU24.1, 18MU24.2, 18MU25.1, 18MU26.1, 18MU27.1, 18MU27.2, 18MU28.1, 18MU29.1, 18MU30.1, 18MU30.2. ND: not done. Vp: villar protrusions of the cyst wall.

**Table 2:** Genetic characterization of *Sarcocystis* spp. detected in wild mountain Caprinae hosts from Spain.

Host	Species	Gene	Haplotypes/*n*	Length (bp)	Values of the Genetic Similarity		
					Compared Between Each Other	Compared to Sequences of the Same Species from GenBank	With Other Related Species Giving Highest Values
Balearean wild goat	*S. cornagliai*	*18S* rRNA	3/3	1874	99.9%	99.9–100%	88.8–89.7% with *S*. *silva*
		*cox1*	3/3	1077	99.8–99.9%	99.9–100%	75.3–75.4% with *S*. *dehongensis*
Pyrenean chamois	*S*. *tenella*	*18S* rRNA	5/15	1828–1829	99.8–100%	96.8–100%	98.3–99.4% with *S*. *capracanis*
		*cox1*	17/17	1005–1029	98.6–99.9%	95.5–100%	91.7–93.9% with *S*. *capracanis*
Mouflon	*S*. *tenella*	*18S* rRNA	5/23	1828–1829	99.8–100%	96.8–100%	98.3–99.4% with *S*. *capracanis*
		*cox1*	23/23	907–1029	98.7–99.9%	95.6–100%	90.0–94.5% with *S*. *capracanis*
Iberian ibex	*S*. *capracanis*	*18S* rRNA	14/22	1823–1827	98.7–100%	97.9–100%	96.3–99.5% with *S*. *tenella*
		*cox1*	21/24	1029	97.9–100%	97.3–100%	91.4–93.6% with *S*. *tenella*
	*S*. *rossii*	*18S* rRNA	1/1	1831	-	100%	96.4–97.4% with *S*. *arieticanis*
		*cox1*	1/1	1029	-	98.4–98.9%	86.2–86.7% with *S. hircicanis* 81.4–86.7% with *S. arieticanis*

*n*—total number of sequences.

## Data Availability

The sequences of *Sarcocystis* spp. were submitted to the NCBI GenBank database under accession numbers PZ406984–PZ407047, PZ436359–PZ436426.

## Supplementary Materials

The following supporting information can be downloaded at https://www.mdpi.com/article/10.3390/pathogens15080816/s1, Figure S1: Phylogenetic subtrees derived from *cox1* sequence analysis showing clustering of *S*. *tenella* (A) and *S*. *capracanis* (B) detected in wild Caprinae host species from Spain; Table S1: Reports of *Sarcocystis* spp. infecting mountain ungulates in Europe.

## Abbreviations

The following abbreviations are used in this manuscript:

TEM	Transmission electron microscopy
DH	Definitive host
IH	Intermediate host
*18S* rRNA	Ribosomal RNA small unit
*Cox1*	Mitochondrial c oxidase 1 unit
vp	Villar protrusion
