# Supplementary material for: Sarcocystis spp. Investigations in Iberian Wild Caprinae Reveal a Remarkable Degree of Interconnection with Parasites’ Domestic Epidemiological Life Cycles
Source: Pathogens. 2026 Aug 1;15(8):816. doi: 10.3390/pathogens15080816 (PMC13516470; doi:10.3390/pathogens15080816)
Supplement: Supplementary file 1 [file pathogens-15-00816-s001.zip › Table S1.pdf]

Table S1: Reports of *Sarcocystis* spp. infecting mountain ungulates in Europe.

Species IH	Country	*Sarcocystis* spp. (Occurrence, %)	Observations and Cyst wall morphology	Methods	References
**Chamois** (*Rupicapra rupicapra*)	Italy	79.8% (158/198) Myocardium solely. Likely *S. ovicanis* (*tenella*) in myocardium.	Palisade-digitiform VP, several sloped or parallel to CW.	LM, TEM	[3]
		Sarcocystis sp. *cf. bovifelis* (*hirsuta*) or *S. bovihominis* (*hominis*) in diaphragm.	Pedunculated or mushroom VP with electrondense fibrils in the core.		
		80.4% (37/46) *Sarcocystis* spp. Myocardium solely.	Ovoid sarcocysts with radiate CW	LM	[4]
	Austria and Germany	*S. cornagliai, Sarcocystis* spp. ND on prevalence or number of animals.	CW type 24 mushroom VP, CW type 14 finger-like VP, respectively.	LM, TEM, SEM	[5]
	Spain	*Sarcocystis* spp. 19% (10/52)	In a study of sarcoptic mange. Heart and intercostal muscles. ND on morphology.	LM	[6]
		Sarcocystis spp. 20 animals studied, ND on prevalence or number of positive animals.	In a study of Border disease infection. Skeletal and hearth muscles. ND on morphology	LM	[7]
		Sarcocystis spp. 100% (6/6)	ND on morphology.	Trypsin digestion, LM	[8]
	Slovak Republic	*Sarcocystis* spp. ND. 65% (32/49)	ND	LM	[9]
	Poland	ND. *Sarcocystis* spp. 77.3% (167/216)	ND	LM	[10]
**Tatra chamois** (*Rupicapra rupicapra tatrica*)	Poland	*S. tenella,* 100% (3/3)	Thin CW apparently without VP	LM, Molecular analyses (***18S rDNA, cox1***)	[11]
**Mouflon** (*Ovis aries*	Italy	*S. sp*. 100 % (6/6)	Similar to *S. tenella* (Finger-like VP).	LM, TEM, pepsin digestion, dogs & cats feed with infected muscles	[12]

Species IH	Country	*Sarcocystis* spp. (Occurrence, %)	Observations and Cyst wall morphology	Methods	References
musimon)	Germany	*S. tenella* 100% (8/8); *S. arieticanis* 100% (8/8)	Finger-like VP. Hair-like VP respectively.	LM, TEM	[13]
		Sarcocystis spp. 90.2% (37/41)	ND on *Sarcocystis* spp. CW could not be observed after trypsin digestion	Trypsin digestion.	[14]
		S. gigantea 33.3% (1/3).	One mouflon (zoo kept). Semi-spheric cyst about 1 cm length, CW with bulbous VP	Direct observation, LM	[10]
	Slovak Republic	*Sarcocystis* spp. 100% (10/10).	ND on morphology.	Trypsin digestion, LM to obtain *Sarcocystis* density	[8]
	Spain	*Sarcocystis* spp.	At genus level. Only announce its presence in this IH. ND on morphology	Literature review	[15]
	Austria	*S. tenella*, 94.7% (18/19)<br>*S. arieticanis*, 5.3% (1/19)<br>*S. capracanis* 5.3% (1/19)<br><br>In digestion:95% (19/20)	Thick CW with finger-like VP at LM	LM, pepsin digestion<br><br>Molecular analyses (***cox1***).	[16]
**Iberian ibex** (*Capra pyrenaica*)	Spain	*S. capricornis*-like 100% (1/1)	*S. capricornis-like,* CW type 41, Digitiform VP with indented edges fire flames-like	LM, TEM	[17]
		Sarcocystis spp. 27.4% (118/430)	At genus level. Only announce its presence in this IH. ND on morphology	Literature review	[18]
		Sarcocystis spp. 23.5% (12/51)	In a study of horn quality. ND on morphology	LM	[19]
		Sarcocystis spp. 32% (14/43)	In a study of sperm quality. ND on morphology	LM	[20]
		Sarcocystis spp. 84% (42/50)	Thin CW and smooth, tentatively *S. hircicanis*	LM, trypsin digestion	[21]

Species IH	Country	*Sarcocystis* spp. (Occurrence, %)	Observations and Cyst wall morphology	Methods	References
**Alpine ibex** (*Capra ibex*)	Italy	Overall: 86% (47/55)<br>*S. cornagliai* 27% (15/54)<br>*S. capracanis* 53% (29/54)<br>*S. arieticanis* 3% (2/54)	*S. cornagliai*, thick CW type 24, mushroom VP, microfilaments in the central area penetrating GS<br>*S. capracanis,* thick CW type 14, elongated VP in villus-like formation with variable length<br>*S. arieticanis*, thin CW elongated digitation of variable length and discontinuous VP parallel to CW, without inner fibrillar elements	LM, TEM	[17]
		80.5% (33/41) *Sarcocystis* spp. Myocardium solely.	Ovoid sarcocysts with radiate CW	LM	[4]
	Austria	*S. capracanis*<br>*S. cornagliai*<br><br><br>*S. rossii* n. sp.	Thick CW type 14, finger-like VP<br>Thick CW type 24, mushroom- or finger-like vp with dense core of MT in central area penetrating GS<br>Thin CW type 7a, hair-like VP with dome-shape at the base	LM, TEM<br><br>Molecular analyses (*18S rRNA* & ***cox1***)	[22]
**Barbary sheep** (*Ammotragus lervia*)	Spain	Global 33.9% (19/56)<br>*S. capracanis*<br>*S. tenella*<br><br>Likely *S. hircicanis*/*S. arieticanis*	*S. capracanis* and *S. tenella,* thick CW type 14, Digitiform VP<br><br>Likely *S. hircicanis/S. arieticanis,* thin CW type 7b, dome-shape base and thread or finger-like VP	LM,TEM<br><br>Molecular analyses (*18S rRNA* & ***cox1***)	[2]

ND: no data. LM: light microscopy. MT: microtubules. Genes used in molecular methods in bold letter.
